# Impact of resuscitation with 100% oxygen during physiological-based cord clamping or immediate cord clamping on lung inflammation and injury

**DOI:** 10.1038/s41390-025-04285-6

**Published:** 2025-08-15

**Authors:** Zoe Poulos, Emma Vandenberg, Zoe Johnson, Valerie A. Zahra, Hui Lu, Alison Thiel, Stuart B. Hooper, Ebony R. Cannata, Shiraz Badurdeen, Robert Galinsky, Nhi Tran, Georg M. Schmölzer, Arjan te Pas, Andrew W. Gill, Martin Kluckow, Calum T. Roberts, Graeme R. Polglase

**Affiliations:** 1 The Ritchie Centre, Hudson Institute of Medical Research, Clayton, VIC Australia; 2 Department of Paediatrics, Monash University, Clayton, VIC Australia; 3 Department of Obstetrics and Gynaecology, Monash University, Clayton, VIC Australia; 4 Department of Paediatrics, Mercy Hospital for Women, Heidelberg, VIC Australia; 5 Department of Pediatrics, University of Alberta, Edmonton, AB Canada; 6 Centre for the Studies of Asphyxia and Resuscitation, Neonatology, Royal Alexandra Hospital, Edmonton, AB Canada; 7 Division of Neonatology, Department of Pediatrics, Leiden University Medical Center, Leiden, The Netherlands; 8 Centre for Neonatal Research and Education, The University of Western Australia, Subiaco, WA Australia; 9 Department of Neonatology, Royal North Shore Hospital & University of Sydney, Sydney, NSW Australia; 10 Monash Newborn, Monash Children’s Hospital, Clayton, VIC Australia

## Abstract

**Background:**

We examined whether physiological based cord clamping (PBCC) reduces oxygen-induced lung inflammation compared to immediate cord clamping (ICC) in preterm lambs ventilated with 100% oxygen for 10 min after birth.

**Methods:**

Instrumented, steroid exposed preterm lambs (125 ± 1 days’ gestation) were randomized to receive 10 min of resuscitation with 100% oxygen before umbilical cord clamping or 30 s after ICC. After 10 min, oxygen was titrated to target SpO_2_ of 90–95%. At 1 h, the lungs were collected for analysis of oxidative stress and inflammation, including RNASeq, and compared to an unventilated control group (UVC).

**Results:**

PBCC prevented the transient fall in SpO_2_ caused by ICC and reduced PaO_2_, pulmonary blood flow, mean and diastolic blood pressure during 100% oxygen ventilation. Lung markers of oxidative stress and inflammation were increased in PBCC and ICC lambs compared to UVC (all *p* < 0.01). IL1ß and IL6 gene expression was higher in PBCC than ICC lambs. Transcriptome analysis revealed novel pathways related to inflammation, immune response, and cytokine signalling.

**Conclusion:**

PBCC prevents initial hypoxia and subsequent hyperoxia from 100% oxygen ventilation, but it does not reduce lung oxidative stress, inflammation, or injury, nor the risk or severity of lung damage during high-oxygen resuscitation.

**Impact:**

- The appropriate oxygen level to use during the initial resuscitation of preterm infants during delayed cord clamping/physiological based cord clamping is not known.
- We found that providing prolonged high oxygen during physiological based cord clamping to preterm lambs does not protect the preterm lung from inflammation, injury or oxidative stress compared to immediate cord clamping.
- The use of prolonged high oxygen during the initial resuscitation of preterm newborns should be avoided irrespective of what cord clamping strategy is being used.

## Introduction

Preterm birth remains a leading cause of newborn morbidity and mortality with an estimated 900,000 infants dying annually.^1^ The main cause of mortality is a reduced capacity for gas exchange caused by lung immaturity.^1,2^ As a consequence, preterm infants often require respiratory support to survive after birth. However, in the preterm infant, respiratory support in and of itself causes lung inflammation and injury, which can lead to the development of bronchopulmonary dysplasia with lifelong consequences.^3,4^ Although the causes of ventilation-induced lung injury are multifactorial, a major component is oxygen. Oxygen is vital in the care of extremely preterm infants (<28 weeks’ gestation) and is given more often than any other drug. However, little is known about what oxygen levels are safe, particularly at birth when the lung is liquid-filled or partially liquid-filled. Excessive oxygen causes oxidative stress resulting in the production of reactive oxygen species which damage cellular integrity^5–7^ and triggers an inflammatory cascade, increasing cellular injury.^8^ However, a recent systematic review and individual participant data network meta-analysis demonstrated reduced mortality when preterm (<32 weeks) resuscitation was started in high (>0.9 FiO_2_) oxygen.^9^ Conversely, lower fractions of inspired oxygen (FiO_2_, 0.21–0.30) are currently recommended when initiating respiratory support of preterm infants,^10^ but low FiO_2_ levels increase the risk of mortality and failure to meet oxygen saturation targets compared to starting with high oxygen (0.6–1.0 FiO_2_).^11,12^ As a result, it is unclear what oxygen levels should be used during the initiation of respiratory support, to best prevent hypoxia and increased mortality, while also avoiding hyperoxia and increased morbidity.

Most studies examining and recommending the use of low initial FiO_2_ levels were conducted following immediate umbilical cord clamping (ICC), where the neonate is removed from its primary oxygen source (the placenta) prior to respiratory support commencing, further confounding their interpretation. Clinical trials are investigating the utility of physiological-based cord clamping (PBCC), which is designed to aerate the lung, either by spontaneous breathing or by the provision of respiratory support, prior to cord clamping.^13,14^ Preclinical studies have demonstrated that PBCC improves cardiovascular stability and prevents systemic and cerebral hypoxia.^15,16^ Further, a recent individual patient data meta-analysis reported that DCC > 2 min/PBCC was associated with reduced mortality in extremely preterm infants.^17,18^ However, the ideal starting oxygen during PBCC is not known. Preclinical evidence demonstrates that in ventilated preterm lambs, >0.50 FiO_2_ is required to achieve target oxygen saturations after ICC, but the same targets can be achieved in 0.21 FiO_2_ if PBCC is applied.^16^ Whilst this suggests PBCC may lower oxidative injury, no studies have investigated this contention. Despite this critical knowledge gap, trials are investigating the use of 1.0 FiO_2_ during PBCC,^19^ with little knowledge of whether PBCC provides protection to the preterm lung. We aimed to determine whether PBCC reduces pulmonary oxidative stress compared to ICC, if applied in combination with 10 min of respiratory support with 1.0 FiO_2_. We hypothesised that PBCC would reduce oxidative stress, lung inflammation and injury compared to ICC in preterm lambs.

## Methods

Experimental methods were approved by Monash Medical Centre-A Animal Ethics Committee (approval number 2023/05) and were conducted in accordance with the National Health and Medical Research Council (NHMRC) guidelines.^20^

### Instrumentation and delivery

Singleton bearing pregnant ewes (Border Leicester) at 123 ± 1 days’ gestation (d; term ~148 days) received four intramuscular doses of dexamethasone (6 mg dexamethasone [Dexamethasone Phosphate; Mylan, Pennsylvania] in 12-h intervals (48–12 h) prior to surgery. At this gestation, the lungs are functionally equivalent to a 26 week preterm infant.^21^ At 125 ± 1 d, ewes were anaesthetised with intravenous thiopentone (20 mg/kg) and inhaled isoflurane (1.5–2.5% in room air/oxygen) and the fetus was exteriorised via caesarean section. The fetus was instrumented with catheters in the left jugular vein and carotid artery and flow probe around the left main pulmonary artery (4–6 mm, Transonics) as previously described.^22,23^ The fetus was intubated with a cuffed endotracheal tube, initially clamped to prevent lung liquid drainage prior to delivery. A transcutaneous oximeter was attached to the right forelimb for measurement of oxygen saturation (SpO_2_; Masimo SET Pulse Oximeter; Masimo, California). Immediately prior to surgery lambs were randomly allocated using a web-based randomiser (www.random.org/lists) to an experimental group:

1. Unventilated control (CTRL, *n* = 8): lambs were delivered immediately euthanised with no instrumentation or ventilation;
2. Immediate cord clamping (ICC, *n* = 7): lambs underwent ICC following delivery, then received 10 min of 1.0 FiO_2_ during ventilation. Respiratory support began 30 s after umbilical cord clamping;
3. Physiological-based cord clamping (PBCC, *n* = 7): respiratory support was provided with 1.0 FiO_2_ for 10 min prior to umbilical cord clamping.

ICC and PBCC lambs were delivered, dried, and commenced on endotracheal positive pressure ventilation in volume guarantee mode, targeting a tidal volume of 7 ml/kg (Drager 8000+; Drager, Lübeck, Germany) as this is the normal tidal volume achieved by preterm lambs spontaneously breathing on CPAP.^24^ Lung liquid was drained immediately prior to the commencement of ventilation. Lambs were not given surfactant in this study. After 10 min of ventilation with 1.0 FiO_2_, lambs were moved from the ewe to an infant warmer and ventilated for 50 min. FiO_2_ was adjusted to maintain target arterial oxygen saturations (90–95%) while rate and tidal volume were adjusted to allow permissive hypercapnia. Arterial and venous blood gases were collected during the experiment to assess lamb wellbeing and guide ventilation parameters (ABP30; Radiometer, Copenhagen, Denmark). The lamb remained anaesthetised throughout the study via an Alfaxan infusion to prevent spontaneous breathing (5–15 mg/kg/h, Alfaxan; Jurox, New South Wales, Australia). Both ewe and lamb were euthanised with an overdose of intravenous phenobarbitone at the experiments’ conclusion (100 mg/kg IV, Lethobarb; Virbac, New South Wales, Australia).

### Lung collection

Lungs were collected immediately post-mortem. Small random pieces were collected from the left lung and snap-frozen in liquid nitrogen, while the right lung was perfusion-fixed with 10% formalin at 20 cmH_2_O. The upper lobe was sectioned into 0.5 cm slices, with three 1.5 cm^2^ sections randomly selected for immersion in Zamboni’s fixative solution for 24 h. Sections were processed and mounted into paraffin wax blocks, before being microtomed into 5-micron thick sections and mounted onto glass slides.^25^

### Histological analysis and quantification

Tissue was stained with haematoxylin and eosin (H&E) to assess lung morphology. Sections were immersed in oil and imaged at ×100 magnification (H&E). Five random, non-overlapping fields of view (excluding major airways or blood vessels) were captured of each section (Olympus DP27 Colour Camera; Olympus, Tokyo, Japan). Sections were used to quantify the mean linear intercept and percentage of lung tissue and airspace volumes in each image, as described by the American Thoracic Society.^26^

### Immunohistochemical analysis and quantification

Leucocytes were visualised using CD45 (Mouse anti Sheep CD45, 1:200 dilution; Bio Rad, California, #MCA896GA), nucleic acid oxidation was visualised using 8-hydroxy-2’-deoxyguanosine (8-OHdG; Mouse anti 8-OHdG, 1:2000 dilution; Abcam, Oregon, #ab48508) and peroxidase enzyme activity assessed using myeloperoxidase (MPO; Rabbit anti Human MPO, 1:500 dilution, Agilent (Dako), Santa Clara, Cat# A039829-2) immunohistochemistry. Heat-mediated antigen retrieval was performed (CD45, 0.1 M sodium citrate buffer, pH 6; 8-OHdG, Target Retrieval Solution, High pH, DAKO; MPO, Proteinase K 1:500) prior to primary antibody incubation (CD45 and MPO, overnight at 4 °C; 8-OHdG, 60 min at room temperature). Sections then underwent secondary antibody incubation (CD45, Goat anti Mouse IgG, 1:200 dilution: Vector Laboratories, California, #BA-9200-1.5; 8-OHdG, HRP anti Mouse4 IgG; DAKO, Glostrup, Denmark, # K4001; MPO, biotinylated Goat anti Rabbit IgG, 1:200 dilution, Vector Laboratories, California, #BA100) before reacting with 3,3’-Diaminobenzidine complex. Sections were counterstained with haematoxylin, dehydrated in ethanol, cleared in xylene and cover slipped. Stained CD45 and 8-OHdG cells were manually counted in each image and expressed as mean number of positive cells per section. Total cell count was derived from CD45 stained sections, using Fiji ImageJ processing software (v2.14.0, LOCI, Wisconsin).

### Molecular analysis

RNA was extracted from lung tissue (RNeasy Maxi Extraction Kit; Qiagen, Hilden, Germany) and reverse transcribed into cDNA (SuperScript III reverse transcriptase; Invitrogen, California). cDNA samples were submitted to the MHTP Genomics Facility (Hudson Institute of Medical Research; Clayton, Australia) where they underwent high-throughput RT-qPCR using Fluidigm® Access Array System technology (Fluidigm® Corporation, California) using TaqMan primers (Thermo Fisher Scientific, Massachusetts). mRNA levels of oxidative stress genes (*NOS1*, *NOS2*, *NOS3*, *NOX1*, *NOX2*, *SOD2*, *GPX1*, *CAT*, *NRF1*, *UCP2*) and inflammatory genes (*IL-1A*, *IL-1B*, *IL-6*, *IL-8*, *IL-10*, *PTGS2*, *HSP70*) were determined (Table 1). Levels of mRNA in each group are expressed as a fold-change from the unventilated controls. Gene expression was normalised to gene ribosomal protein 18 (*RPS18*), a reference gene with stable mRNA levels in sheep.^27^

**Table 1:** Details of genes included for molecular analysis.

Gene category	Gene name & abbreviation		TaqMan assay ID
Reference gene	Ribosomal protein S18	*RPS18*	Oa4906333_g1
Markers of oxidative stress	Nitric oxide synthase 1	*NOS1*	Oa04714367_m1
	Nitric oxide synthase 2	*NOS2*	Oa04876175_m1
	Nitric oxide synthase 3	*NOS3*	Oa04907031_MH
	NADPH oxidase 1	*NOX1*	Oa04709255_g1
	NADPH oxidase 2	*NOX2*	Oa04793417_m1
Antioxidants	Superoxide dismutase 2	*SOD2*	Oa04657474_m1
	Glutathione peroxidase 1	*GPX1*	Oa04911462_g1
	Catalase	*CAT*	Oa03228713_m1
	Nuclear respiratory factor 1	*NRF1*	Oa04660399_m1
	Uncoupling protein 2	*UCP2*	Oa03225185_g1
Inflammatory cytokines	Interleukin 1 alpha	*IL-1A*	Oa04658682_m1
	Interleukin 1 beta	*IL-1B*	Oa04656322_m1
	Interleukin 6	*IL-6*	Oa04656315_m1
	Interleukin 8	*IL-8*	Bt03211906_m1
	Interleukin 10	*IL-10*	Oa03212724_m1
Inflammatory mediators	Prostaglandin-endoperoxide synthase 2	*PTGS2*	Oa04657348_g1
	Heat shock protein 70	*HSP70*	Oa04849683_g1

Full names of genes with corresponding abbreviations, categories and TaqMan Assay IDs. Assay IDs are provided in lieu of primer sequences, as these are not publicly available.

### Total RNA extraction and sequencing

Lamb lung RNA was isolated and DNase treated using a RNeasy Maxi kit (Qiagen; Hilden, Germany) as per manufacturer’s protocol and eluted in RNase-free distilled water and stored at −80 °C until further analysis.

RNA samples were submitted to the MHTP Genomics Facility (Hudson Institute of Medical Research; Clayton, Australia) for integrity and concentration assessment via capillary electrophoresis (Agilent Technologies) and fluorometric quantification (Qubit, Invitrogen). RNA integrity number (RIN) was ≥7.5 for all samples, so considered viable for RNA-sequencing analyses.

RNA-Seq was then performed using a custom in-house multiplex method similar to that previously described (1–3). Briefly, samples were given a unique i7 index (together with UMI) during individual pA priming and first strand synthesis which also adds a template switch sequence to the 5’-end. Samples were then pooled into sets and amplified using P7 and an oligo which binds the template switch sequence. Final library construction was completed by tagmentation and addition of P5 by PCR. Sequencing was performed on an Illumina NSQ2k run with 111nt SR (cDNA). A 20nt i7 read contains the 10nt index and 10nt UMI. Samples were parsed using unique i7 indexes.

### Data analysis and bioinformatics

Data analysis and bioinformatics were performed at the Monash Bioinformatics Platform (Clayton, Australia). The nf-core/rnaseq Nextflow pipeline version 3.10.1^28^ was used to process the RNA sequencing reads. ENSEMBL version 111 Sheep (*Ovis Aries*) was used as the reference genome. Briefly, reads are trimmed using Trim Galore^29^ and then aligned to the genome using STAR.^30^ STAR produces alignments to transcripts that are then deduplicated using UMI-tools^31^ and quantified using Salmon.^32^ Genes with fewer than 10 UMIs in at least one sample were filtered. Differential gene expression analysis was then carried out at the gene level with the Degust web application^33^ using the limma-voom method.^34^

Over-representation analysis (ORA) of differentially expressed genes in gene sets was then performed using the clusterProfiler package.^35^ Genes were considered differentially expressed if they had an estimated fold-change of at least 2-fold and an FDR less than 0.05. Gene sets considered were taken from gene ontology (GO) and Kyoto Encyclopedia of Genes and Genomes (KEGG). Gene sets were used if they contained at least 10 genes, not including filtered genes.

### Physiological analysis

The required sample size of 6–8 lambs was calculated using a power analysis on G*Power (3.1.9.7) for the primary outcome of oxidative stress. The power was set to 0.8, and the type one error rate to 0.05.

Physiological data, including heart rate, pulmonary blood flow, blood pressure and systemic oxygen saturation, was collected and analysed using LabChart software (ADInstruments, New South Wales, Australia). Blood gas data were combined with FiO_2_ to calculate the partial the Alveolar-arterial (A-a) gradient.^36^

### Statistical analysis

Investigators were blinded to interventions during data analysis. All data are presented as mean ± standard deviation (SD) and was analysed using GraphPad Prism (GraphPad Software, California). Normality was assessed using a Shapiro–Wilk test. Fetal characteristics, histological/immunohistochemical data and molecular data were analysed using a one-way ANOVA with Tukey’s multiple comparisons test or Kruskal–Wallis and Dunn’s test if data was not normally distributed. Blood gases, ventilation parameters and physiological data were analysed using a two-way ANOVA with repeated measures (experimental group and time as independent factors) and a post hoc Šídák’s multiple comparisons test. If data were not normal, a mixed effects model with a post hoc Šídák’s test was used. Statistical tests analysing blood gases, FiO_2_ and physiological data were analysed separately over the first 10 min and final 50 min of the experiment. Significance was accepted when *p* < 0.05.

## Results

### Baseline characteristics

Twenty-three lambs were included in the analysis and remained viable until the end of the experiment. Baseline group and arterial blood gas characteristics are outlined in Table 2. Mean gestational age and mean body weight were significantly lower in the ICC lambs compared to the control lambs. No other differences in baseline characteristics or blood gas variables were observed between groups.

**Table 2:** Baseline fetal and arterial blood gas characteristics.

	Control	ICC	PBCC
Group characteristics			
Number (*n*)	8	7	7
Sex (% Male)	38	43	88
GA (days)	126 **± **1	124* **± **1	125 **± **1
Body weight (kg)	3.5 **± **0.5	3.0* **± **0.2	3.23 **±** 0.3
Lung weight (g)	111.5 **± **30.5	99.7 **±** 17.1	116.6 **± **18.5
Baseline arterial blood gas characteristics			
pH	7.30 **±** 0.09	7.19 **± **0.20	7.24 **±** 0.10
PaCO_2_ (mmHg)	59.9 **±** 14.4	65.5 **±** 17.6	56.6 **±** 7.8
PaO_2_ (mmHg)	25.3 **±** 13.5	18.3 **±** 4.9	23.5 **±** 5.9
SaO_2_ (mmHg)	53.84 **± **29.4	43.1 **±** 24.3	59.09 **± **21.9
Ventilation parameters			
PIP (cmH_2_O; mean first 10 min)	–	35.7 **±** 3.3	33.6 **±** 2.6
Paw (cmH_2_O; mean first 10 min)	–	18.8 **±** 1.8	17.8 **±** 1.3
Tidal volume (ml/kg; mean first 10 min)	–	6.4 **±** 2.0	6.3 **±** 1.8
Total protein content (Mean **± **SD)	673.0 **± **162.5	1069.0 **± **218.6*	1054.0 **± **231.5*

Data are presented as mean **± **SD unless otherwise indicated.

*PIP* peak inflation pressure, *Paw* mean airway pressure measured during the first 10 min of ventilation.

*p < 0.05 vs. Control.

### Ventilation parameters

Ventilation parameters, including tidal volume, peak inflation pressure and mean airway pressure were not different between groups during the first 10 min (Table 2), or thereafter.

### Blood gas status and oxygenation

**Fig. 1:**
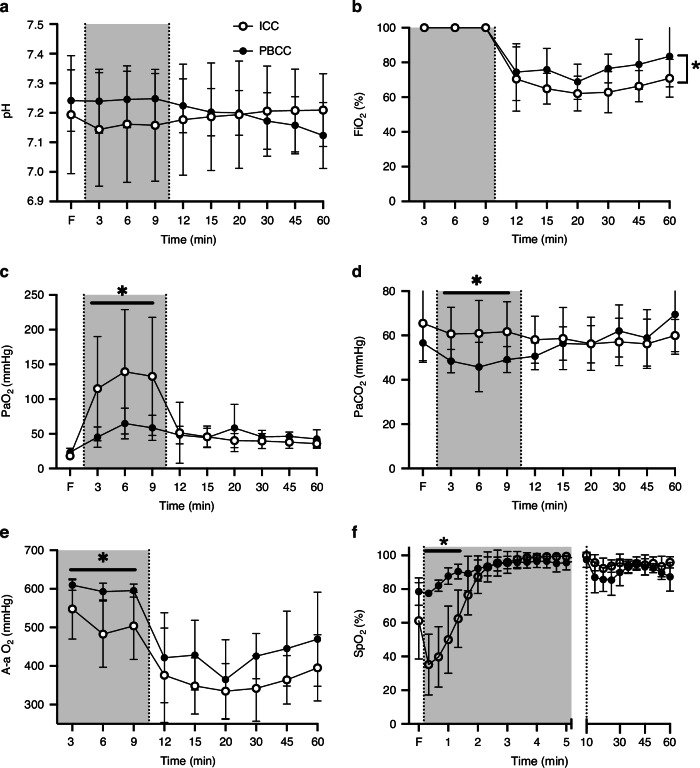
Arterial blood gas and oxygenation. **a** pH, **b** FiO_2_, **c** PaO_2_, **d** PaCO_2_, **e** Alveolar-arterial oxygen gradient (A-a O_2_), and **f** SpO_2_ measured immediately prior to ventilation (F, Fetal), during the first 10 min of 100% oxygen (shaded area) and subsequent 50-min ventilation period in physiological-based cord clamping (black circles) and immediate cord clamping (open circles) lambs. *p < 0.05. Data are mean ± SD.

pH was not different between groups throughout the study (Fig. 1). FiO_2_ requirement was significantly higher in PBCC lambs compared to ICC after 10 min (*p* = 0.028). The partial pressure of arterial oxygen (PaO_2_, *p* = 0.032) and arterial carbon dioxide (PaCO_2_, *p* = 0.020) were significantly higher in ICC lambs compared to PBCC lambs over the first 9 min of resuscitation (Fig. 1). The A-a O_2_ gradient was higher in PBCC lambs during the first 9 min of resuscitation (*p* = 0.031) compared with ICC lambs but was not different thereafter. PBCC prevented the rapid hypoxia that occurs with cord clamping prior to lung aeration, with significantly higher SpO_2_ during the first 90 s of ventilation (*p* = 0.01); SpO_2_ was not different between groups thereafter. All blood gas parameters were not different between groups after 10 min.

### Physiological data

**Fig. 2:**
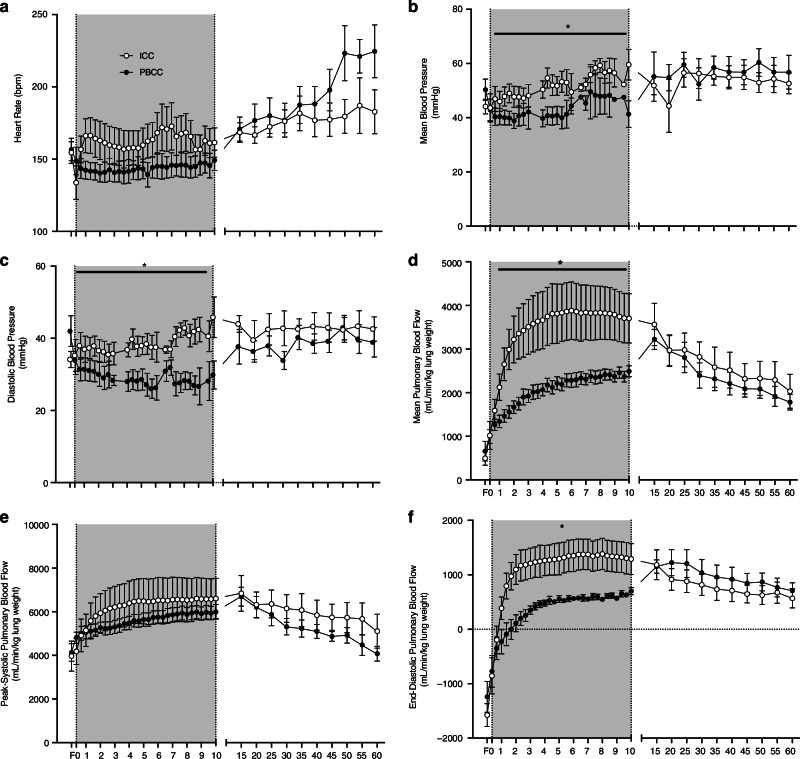
Physiological parameters. **a** Heart rate, **b** mean blood pressure, **c** diastolic blood pressure, **d** mean pulmonary blood flow, **e** peak-systolic pulmonary blood flow and **f** end-diastolic pulmonary blood flow recorded during the first 10 min of 100% oxygen (shaded area) during physiological-based cord clamping (black circles) and after immediate cord clamping (open circles) and subsequent 50-min ventilation period. *p < 0.05. Data are mean ± SEM.

Heart rate was significantly higher in ICC lambs over the first 10 min of the experiment compared to PBCC lambs (*p* = 0.0156; Fig. 2). Heart rate was not different between groups from 15 min.

Mean and diastolic blood pressure were higher in ICC lambs (*p* = 0.025 and *p* = 0.017, respectively; Fig. 2) compared to PBCC lambs during the first 10 min of resuscitation, but not thereafter. Systolic blood pressure was significantly higher in ICC lambs between 4–6 min and 8–9 min (*p* < 0.05 for all; data not shown) compared to PBCC lambs, but not thereafter.

Mean and end-diastolic pulmonary blood flow were higher in ICC lambs during the first 10 min of resuscitation compared to PBCC lambs (*p* = 0.0130 and *p* = 0.0154, respectively; Fig. 2). There were no significant differences in mean or end-diastolic pulmonary blood flow between the groups for the remainder of the experiment. Peak-systolic pulmonary blood flow was similar between groups for the entire experiment.

### Histological lung inflammation, injury and oxidative stress

**Fig. 3:**
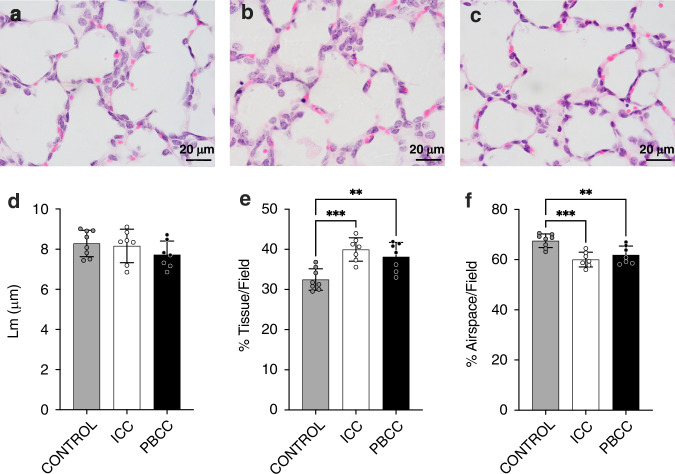
Lung histology. **a**–**c** Representative H&E-stained lung images from control, immediate cord clamping and physiological-based cord clamping lambs, respectively, **d** mean linear intercept (L_m_), **e** % tissue and **f** % airspace. Individual data points plotted with mean ± SD. **p < 0.01, ***p > 0.001. ICC immediate cord clamping (*n* = 7), PBCC physiological-based cord clamping (*n* = 7), control (*n* = 8).

**Fig. 4:**
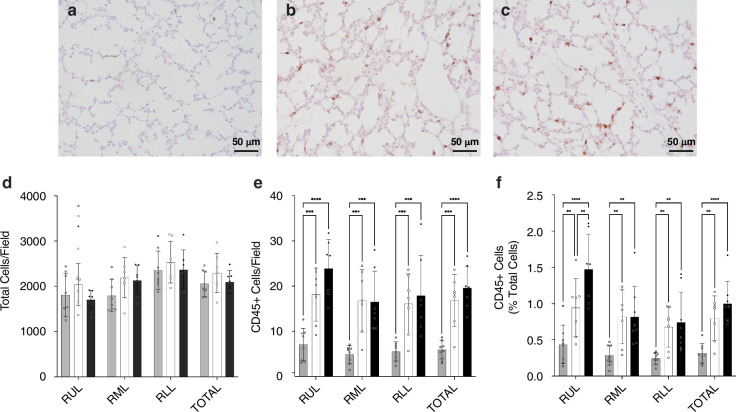
Lung inflammation. **a**–**c** Representative CD45 stained lung images from control, immediate cord clamping and physiological-based cord clamping lambs, respectively, **d** hypercellularity, **e** CD45+ cells and **f** % CD45 in control (grey, *n* = 8), immediate cord clamping (ICC; white; *n* = 7) and physiological-based cord clamping (PBCC, dark grey; *n* = 7) lambs. Data was analysed using a one-way ANOVA with a post hoc Tukey’s test. Individual data points plotted with mean ± SD. **p < 0.01, ***p > 0.001.

The percentage of tissue per field was higher (*p* = 0.0010) and percentage of airspace per field was lower (*p* = 0.0010) in ICC lambs compared to control lambs, but not different to PBCC lambs. Mean linear intercept (L_m_) was not different between groups (Fig. 3). The total number of cells per field of view was not different between groups. ICC and PBCC lambs had increased levels of positive CD45 cells in each of the lung regions examined compared to control lambs (Fig. 4). When adjusted for total number of cells, both ICC and PBCC lambs had an increased proportion of positive CD45 cells relative to total cell count compared to controls with PBCC being higher than ICC lambs within the right upper lobe. Total protein content of the lung was significantly higher in ICC and PBCC lambs compared to controls (Table 1).

Oxidative stress, as determined by the histological assessment of 8-OHdG and Myeloperoxidase positive cells, was not different between groups (Data not shown).

### Gene analysis

#### Oxidative stress

**Fig. 5:**
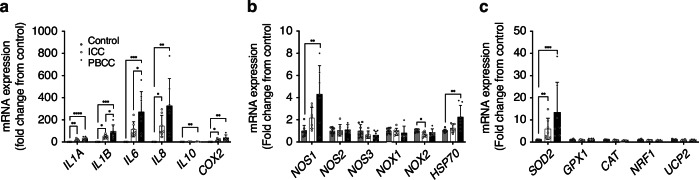
Lung mRNA expression of oxidative stress, antioxidants and inflammatory genes. **a** Inflammatory genes interleukin (IL)-1A, IL-1B, IL-6, IL-8, IL-10 and cyclooxygenase 2 (COX2), **b** oxidative stress genes nitric oxide synthase (NOS)1, NOS2, NOS3, NADPH oxidase (NOX)1, NOX2 and heat-shock protein 70 (HSP70), and **c** antioxidant genes superoxide dismutase 2 (SOD2), glutathione peroxidase 1 (GPX1), catalase (CAT), nuclear respiratory factor 1 (NRF1) and uncoupling protein 2 (UCP2) and in control (grey, *n* = 8), immediate cord clamping (ICC; white; *n* = 7) and physiological-based cord clamping (PBCC, dark grey; *n* = 7) lambs. *p < 0.05, **p < 0.01, ***p < 0.001. Data are mean **±** SD with individual data points shown.

The mRNA expression of pro-oxidant and antioxidant enzymes was assessed (Fig. 5). NOS1 and HSP70 mRNA expression were increased in PBCC lambs (*p* = 0.0022 and *p* = 0.0040, respectively) compared to control lambs while NOX2 mRNA expression was decreased in ICC lambs compared to control lambs (*p* = 0.0173). There were no differences in mRNA expression of NOS2, NOS3 or NOX1 between the groups. PBCC and ICC lambs had increased mRNA expression of SOD2 compared to control lambs (*p* = 0.0004, *p* = 0.0140 respectively). There were no differences in other antioxidant gene expression, including GPX1, CAT, NRF1 or UCP2 between the groups.

### Inflammation

Lung inflammation was assessed by analysis of mRNA expression of pro-inflammatory cytokines (Fig. 5). PBCC and ICC lambs had increased mRNA expression of interleukin (IL)-1A (*p* < 0.0001 and *p* = 0.0083, respectively), IL-1B (*p* < 0.0001 and *p* = 0.0372, respectively), IL-6 (*p* = 0.0004 and *p* = 0.018, respectively), IL-8 (*p* = 0.0006 and *p* = 0.014, respectively) and cyclooxygenase-2 (COX-2; *p* = 0.0008 and *p* = 0.01, respectively) compared to control lambs. PBCC lambs had higher mRNA expression IL-1B (*p* = 0.04) and IL-6 (0.042) compared to ICC lambs. PBCC had increased mRNA expression of IL-10 compared to control lambs (*p* = 0.0011).

### Transcriptome profiling of novel pathways

**Fig. 6:**
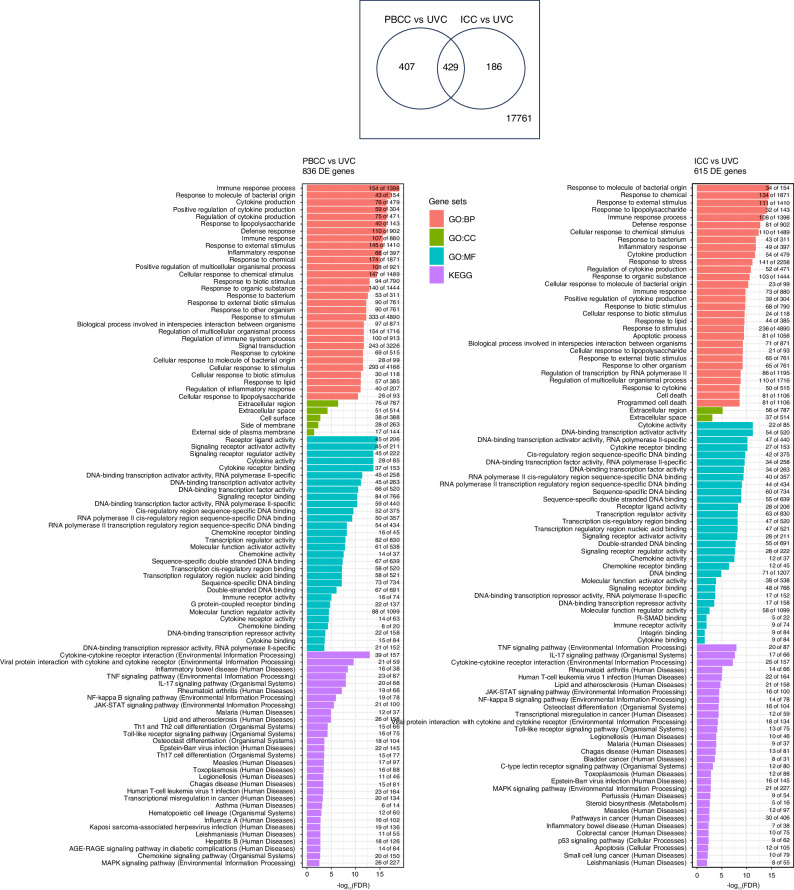
Differential gene expression. Venn diagram depicting the number of differentially expressed genes between PBCC vs. UVC and ICC vs. UVC, noting that no differentially expressed genes were identified between PBCC vs. ICC. Gene ontology (GO) was used to identify the enrichment functions within three categories of genes; biological process (BP; red), cellular component (CC; green) and molecular function (MF; blue), while Kyoto Encyclopedia of Genes and Genomes (KEGG) was used to search for biological pathways (purple).

**Fig. 7:**
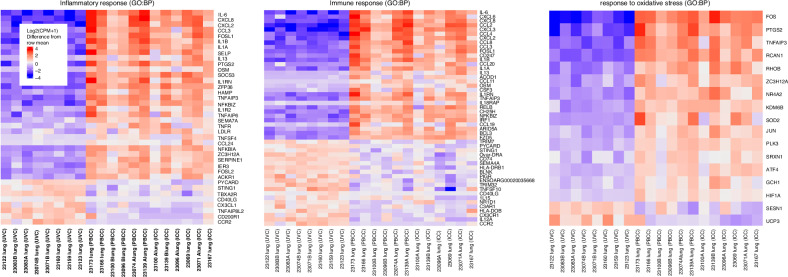
Gene expression of oxidative stress, antioxidants and inflammatory genes. Heat maps developed from the sub-group analysis of the genes identified within the biological processes within the categories of inflammatory response (left panel), immune response (middle panel) and response to oxidative stress (right panel) in lung tissue.

To identify novel pathways induced by high oxygen during respiratory support, RNA-Seq was performed on 8 UVC, 6 ICC and 6 PBCC lamb lungs. We identified 615 differentially expressed genes (up- or down-regulated; FDR < 0.05) between ICC vs. control, and 836 genes between PBCC and control with 429 overlapping genes (Fig. 6). No differentially expressed genes were identified between ICC and PBCC. Subgroup analyses within biological processes, cellular component and molecular function (Gene ontology) and biological processes (KEGG) identified the most prominent differentially expressed genes across inflammation, immune response and oxidative stress (Fig. 7). Biological processes related to inflammation, immune response, cytokine signalling and response to external stimuli were identified, including key pathways of NFKB, JAK-STAT, interleukins (-1, -6, -8, -17, TNF), and COX-2.

## Discussion

Oxygen is critical to preterm newborn care, however, it is not known what the ideal starting FiO_2_ is during the initiation of respiratory support. A recent systematic review showed reduced mortality when FiO2 > 0.9 was used for initial resuscitation of preterm infants <32 weeks’ gestation.^9^ However, two small studies demonstrated that initial high oxygen led to more lung oxidative stress, inflammation and injury when compared to initial low oxygen.^37,38^ But most studies were conducted following immediate cord clamping. It is not known whether physiological-based cord clamping mitigates or amplifies the pulmonary response to high FiO_2_. We investigated whether the provision of 100% oxygen for 10 min during PBCC reduced lung oxidative stress, inflammation and injury compared to ICC lambs. Our study showed that ventilation with 100% oxygen caused significant oxidative stress, inflammation and injury in both cord clamping approaches, with evidence of an amplified inflammatory response in PBCC lambs compared to ICC lambs.

A current clinical trial^19^ is comparing the use of high (100%) vs. low (30%) initial FiO_2_ during delayed cord clamping. Our study was not intended to replicate the clinical scenario whereby FiO_2_ would ideally be rapidly weaned. Rather, it was intended to maximise pulmonary oxidative stress and inflammation by exposing preterm lambs to 10 min of 100% O_2_. As observed previously,^16^ PBCC prevented the rapid fall in SpO_2_ that occurs during the hiatus between umbilical cord clamping and ventilation onset—a fall of ~23% over the first 30–60 s. However, PBCC also prevented systemic hyperoxia, evidenced by the lower PaO_2_ (mean; min-max: 57 mmHg; 30–96 mmHg; vs. 129; 32–265 mmHg) during the 10 min of 100% O_2_ (Fig. 1). This finding is important as high PaO_2_ levels increase the risk of end-organ oxidative stress and tissue injury to multiple organs including the eyes and brain.^39–41^ The finding of higher SpO_2_ at 1 min in PBCC lambs is consistent with our previous finding in preterm lambs receiving 21% O_2_^16^ and indicates that PBCC may allow earlier use of lower FiO_2_ in clinical practice, than is possible with ICC, potentially reducing the overall exposure to high oxygen. Indeed, extremely preterm infants resuscitated with 100% oxygen at birth had higher average oxygen saturation in the first 5 min (85% vs. 58%) than those resuscitated with 30%.^42^ Further, the lower systemic oxygenation in the PBCC may be protective of down-stream organs, particularly the preterm brain. Further analysis of systemic and cerebral oxidative stress, inflammation and injury are needed to confirm this contention.

There are a few potential explanations for the lower PaO_2_ during the first 10 min in PBCC lambs. Firstly, it may be due to reduced pulmonary oxygen diffusion as indicated by the higher A-a O_2_ gradient (Fig. 2). This was likely due to oxygen being retained within the alveoli due to lower pulmonary perfusion, or dilution of left ventricular output due to mixing of pulmonary venous return with umbilical venous blood. PBCC lambs had a slower increase and an overall lower mean pulmonary blood flow during the first 10 min of high oxygen, with continued right-to-left shunting through the ductus arteriosus. This is indicated by a sustained “negative” (retrograde) end-diastolic pulmonary blood flow that was maintained for at least 2 min after ventilation onset. This is indicative of the competition between the lungs and placenta for right ventricular output that has been described previously.^43^ The lower PBF coupled with lower PaO_2_ in PBCC lambs indicates less uptake and removal of oxygen from the lungs, which may create oxygen retention resulting in inflammation and injury. Conversely, Lakshminrumha et al. suggested that during PBCC, oxygenated blood leaving the pulmonary circulation mixes with umbilical blood flow (lower oxygenated) in the left ventricle, decreasing the arterial oxygen content and therefore increasing the A-a O_2_ gradient.^44^ And finally, the reduced PaO_2_ during PBCC may be due to oxygen being lost across the placenta, due reversal of the oxygen gradient. Further investigation on the exact mechanism is needed to determine the cause of the reduced PaO_2_ during PBCC. An interesting finding was the requirement for higher FiO_2_ in PBCC lambs compared to ICC lambs after umbilical cord clamping, which was consistent with observations previously.^15^ The reason for this is not known and requires further investigation.

Contrary to our hypothesis, we did not find any difference in oxidative stress or antioxidant markers (molecular or histological) between ICC and PBCC lambs. Both ventilation strategies increased a number of oxidant and anti-oxidant genes relative to controls (Figs. 5 and 7). Further, nitric oxide synthase 1(NOS1), heat shock protein -70 (HSP-70) and superoxide dismutase 2 (SOD2) were increased in PBCC lambs compared to control, but not ICC lambs. These findings suggest that PBCC is not protective of oxidative stress in the lung receiving high oxygen exposure after birth. Further, another key finding of this study was that PBCC did not reduce lung inflammation or injury resultant from ventilation compared to ICC lambs. We found that ventilation with 1.0 FiO_2_ induced a profound inflammatory response within the airways, as indicated by increased CD45 positive cells in lung tissue and increased airway tissue thickness, compared to unventilated controls (Figs. 3 and 4). Similarly, mRNA expression of pro-inflammatory cytokines IL-1A, IL-1B, IL-6, IL-8 and COX-2 were increased in PBCC and ICC lambs compared to unventilated controls (Fig. 5). These genes are known to be important mediators of ventilation-induced lung injury^25,45,46^ and are associated with BPD pathogenesis in preterm lambs and extremely preterm infants.^47–49^ Importantly, IL-1B and IL-6 were upregulated in PBCC lambs compared to ICC lambs. IL-6 inhibition in neonatal mice reduces hyperoxia-induced lung injury, suggesting this cytokine is critical in oxidative lung injury progression.^50^ PBCC therefore may increase the risk of inflammatory-mediated lung injury than ICC, due to upregulation of IL-1B and IL-6 mRNA, following prolonged 100% O_2_ exposure. Baro- and volu-trauma independently increase proinflammatory cytokines including IL-1B and IL-6 mRNA.^51,52^ Given that mean tidal volumes, airway pressures and peak inflation pressures were not different between groups (Table 1) it is likely our results are due to the physiological differences in cord management strategy, as opposed to ventilatory differences. Taken together, our findings suggest that the sustained (10 min) use of 100% O_2_ at birth should be avoided, irrespective of whether preterm newborns received PBCC or ICC. Clinicians using higher FiO_2_ values during stabilisation at birth should apply particular care to weaning FiO_2_ early, particularly if their oxygen saturation is above target range, increasing the risk for hyperoxia.

A novel aspect of our study was the investigation of pathways of injury resulting from initiation of ventilation in PBCC and ICC lambs (Fig. 6). To our knowledge, this is the first study to thoroughly investigate and compare pathways of injury caused by 100% oxygen ventilation during different cord management strategies. RNASeq identified 429 similar genes up- or down-regulated in both PBCC and ICC groups, with similar biological processes identified between groups. Further interrogation identified important factors well known to increase lung injury, including nuclear factor KB and cytokine pathways.^25,45,46^ Findings from the oxidative stress biological processes are consistent with transcription factors known to be major components of hyperoxic injury, including nuclear factor KB, activator protein 1, nuclear factor, erythroid 2-related factor 2 and signal transducers and activators of transcription protein (STAT) pathways.^53^ Activation of these transcription factors via oxygen is known to increase expression of several genes including IL-8 and IL-6. Importantly, in neonates where alveolar formation relies on postnatal lung development, hyperoxic gene regulation may have detrimental consequences on this normal developmental process resulting in life-long deficits in lung structure and function. These pathways may provide targets for interventions aimed at reducing neonatal hyperoxic injury.

A limitation of the study is that the fetus’s and lambs were anaesthetised throughout the study. The PBCC group were exposed to maternal isoflurane during the initial 10 min whereas the ICC were changed to alfaxane during the initial 10 min. It is not known what influence the different anaesthesias have on the transition at birth, but given that isoflurane has a half-life of 10 min, it is unlikely to have made a significant impact on the study.

## Conclusion

We compared lung oxidative stress, inflammation and injury between PBCC and ICC during 1.0 FiO_2_ for the first 10 min at birth. While PBCC maintained SpO_2_ throughout delivery and reduced systemic PaO_2_, it did not reduce oxidative stress or inflammation in the lung in our study and may amplify some inflammatory pathways. We conclude that sustained high oxygen exposure during resuscitation of preterm newborns, irrespective of the cord management strategy, should be avoided.

## Data Availability

All data, including raw data used for all figures and analysis, and RNASeq data is available upon request to the corresponding author from 3 months following article publication to researchers who provide a methodologically sound proposal for its use. Data requestors will need to sign a material transfer agreement approved by Monash University and the Hudson Institute of Medical Research.

## References

[1] Perin, J. et al. Global, regional, and national causes of under-5 mortality in 2000-19: an updated systematic analysis with implications for the sustainable development goals. *Lancet Child Adolesc. Health* **6**, 106–115 (2022). 34800370 10.1016/S2352-4642(21)00311-4 PMC8786667

[2] Stoll, B. J. et al. Trends in care practices, morbidity, and mortality of extremely preterm neonates, 1993-2012. *JAMA* **314**, 1039–1051 (2015). 26348753 10.1001/jama.2015.10244 PMC4787615

[3] Jobe, A. H. Mechanisms of lung injury and bronchopulmonary dysplasia. *Am. J. Perinatol.* **33**, 1076–1078 (2016). 27603539 10.1055/s-0036-1586107

[4] Doyle, L. W., Ranganathan, S. & Cheong, J. L. Y. Ventilation in preterm infants and lung function at 8 years. *N. Engl. J. Med.* **377**, 1601–1602 (2017). 29045215 10.1056/NEJMc1711170

[5] Auten, R. L., Whorton, M. H. & Mason, S. N. Blocking neutrophil influx reduces DNA damage in hyperoxia-exposed newborn rat lung. *Am. J. Respir. Cell Mol. Biol.* **26**, 391–397 (2002). 11919074 10.1165/ajrcmb.26.4.4708

[6] Luo, X. et al. Effect of the 21-aminosteroid U74389g on oxygen-induced free radical production, lipid peroxidation, and inhibition of lung growth in neonatal rats. *Pediatr. Res.* **46**, 215–223 (1999). 10447118 10.1203/00006450-199908000-00015

[7] Vozzelli, M. A., Mason, S. N., Whorton, M. H., Richard, L. & Auten, J. Antimacrophage chemokine treatment prevents neutrophil and macrophage Influx in hyperoxia-exposed newborn rat lung. *Am. J. Physiol. Lung Cell. Mol. Physiol.* **286**, L488–L493 (2004). 12588706 10.1152/ajplung.00414.2002

[8] Bhandari, V. Hyperoxia-derived lung damage in preterm infants. *Semin. Fetal Neonatal Med.* **15**, 223–229 (2010). 20430708 10.1016/j.siny.2010.03.009 PMC2910132

[9] Sotiropoulos, J. X. et al. Initial oxygen concentration for the resuscitation of infants born at less than 32 weeks’ gestation: a systematic review and individual participant data network meta-analysis. *JAMA Pediatr.* **178**, 774–783 (2024). 38913382 10.1001/jamapediatrics.2024.1848 PMC11197034

[10] Wyckoff, M. H. et al. Neonatal life support: 2020 international consensus on cardiopulmonary resuscitation and emergency cardiovascular care science with treatment recommendations. *Circulation* **142**, S185–S221 (2020). 33084392 10.1161/CIR.0000000000000895

[11] Oei, J. L. et al. Targeted oxygen in the resuscitation of preterm infants, a randomized clinical trial. *Pediatrics* **139**, e20161452 (2017). 10.1542/peds.2016-1452 28034908

[12] Oei, J. L. et al. Outcomes of oxygen saturation targeting during delivery room stabilisation of preterm infants. *Arch. Dis. Child. Fetal Neonatal Ed.* **103**, F446–F454 (2018). 28988158 10.1136/archdischild-2016-312366 PMC6490957

[13] Fairchild, K. D. et al. Ventilatory assistance before umbilical cord clamping in extremely preterm infants: a randomized clinical trial. *JAMA Netw. Open* **7**, e2411140 (2024). 38758557 10.1001/jamanetworkopen.2024.11140 PMC11102017

[14] Knol, R. et al. Physiological versus time based cord clamping in very preterm infants (Abc3): a parallel-group, multicentre, randomised, controlled superiority trial. *Lancet Reg. Health Eur.* **48**, 101146 (2025). 39717227 10.1016/j.lanepe.2024.101146 PMC11664066

[15] Bhatt, S. et al. Delaying cord clamping until ventilation onset improves cardiovascular function at birth in preterm lambs. * J. Physiol.* **591**, 2113–2126 (2013). 23401615 10.1113/jphysiol.2012.250084 PMC3634523

[16] Polglase, G. R. et al. Ventilation onset prior to umbilical cord clamping (physiological-based cord clamping) improves systemic and cerebral oxygenation in preterm lambs. *PLoS ONE* **10**, e0117504 (2015). 25689406 10.1371/journal.pone.0117504 PMC4331493

[17] Seidler, A. L. et al. Deferred cord clamping, cord milking, and immediate cord clamping at preterm birth: a systematic review and individual participant data meta-analysis. * Lancet* **402**, 2209–2222 (2023). 37977169 10.1016/S0140-6736(23)02468-6

[18] Seidler, A. L. et al. Short, medium, and long deferral of umbilical cord clamping compared with umbilical cord milking and immediate clamping at preterm birth: a systematic review and network meta-analysis with individual participant data. *Lancet* **402**, 2223–2234 (2023). 37977170 10.1016/S0140-6736(23)02469-8

[19] Katheria, A. C. et al. Deferred Cord Clamping with High Oxygen in Extremely Preterm Infants: A Randomized Clinical Trial. *JAMA Pediatr.*10.1001/jamapediatrics.2025.2128 (2025). 10.1001/jamapediatrics.2025.2128 PMC12281397 40690234

[20] Council, N. Australian code of practice for the care and use of animals for scientific purposes. Australian Government 2013.

[21] Alcorn, D. G., Adamson, T. M., Maloney, J. E. & Robinson, P. M. A morphologic and morphometric analysis of fetal lung development in the sheep. *Anat. Rec.* **201**, 655–667 (1981). 7340570 10.1002/ar.1092010410

[22] Polglase, G. R. et al. Assessing the influence of abdominal compression on time to return of circulation during resuscitation of asphyxiated newborn lambs: a randomised preclinical study. *Arch. Dis. Child. Fetal. Neonatal Ed.* **109**, 405–411 (2023). 10.1136/archdischild-2023-326047 PMC11228194 38123977

[23] Polglase, G. R. et al. Cardiopulmonary resuscitation of asystolic newborn lambs prior to umbilical cord clamping; the timing of cord clamping matters! *Front. Physiol.* **11**, 902 (2020). 10.3389/fphys.2020.00902 PMC7406709 32848852

[24] Mulrooney, N. et al. Surfactant and physiologic responses of preterm lambs to continuous positive airway pressure. *Am. J. Respir. Crit. Care Med.* **171**, 488–493 (2005). 15502113 10.1164/rccm.200406-774OC

[25] Azman, Z. et al. In utero ventilation induces lung parenchymal and vascular alterations in extremely preterm fetal sheep. *Am. J. Physiol. Lung Cell Mol. Physiol.* **326**, L330–L343 (2024). 38252635 10.1152/ajplung.00249.2023

[26] Hsia, C. C., Hyde, D. M., Ochs, M. & Weibel, E. R. An official research policy statement of the American Thoracic Society/European Respiratory Society: standards for quantitative assessment of lung structure. *Am. J. Respir. Crit. Care Med.* **181**, 394–418 (2010). 20130146 10.1164/rccm.200809-1522ST PMC5455840

[27] Clark, E. L. et al. A high resolution atlas of gene expression in the domestic sheep (Ovis Aries). *PLoS Genet.* **13**, e1006997 (2017). 28915238 10.1371/journal.pgen.1006997 PMC5626511

[28] Patel, H. et al. Nf-Core/Rnaseq: Nf-Core/Rnaseq V3.10.1—Plastered Rhodium Rudolph. *Zenodo* https://zenodo.org/records/7505987 (2023).

[29] Krueger, F., James, F., Ewels, P., Afyounian, E. & Schuster-Boeckler, B. Felixkrueger/Trimgalore: V0.6.7—Doi Via Zenodo. *Zenodo* https://zenodo.org/records/5127899 (2021).

[30] Dobin, A. et al. Star: ultrafast universal RNA-seq aligner. *Bioinformatics* **29**, 15–21 (2013). 23104886 10.1093/bioinformatics/bts635 PMC3530905

[31] Smith, T., Heger, A. & Sudbery, I. Umi-Tools: modeling sequencing errors in unique molecular identifiers to improve quantification accuracy. *Genome Res.* **27**, 491–499 (2017). 28100584 10.1101/gr.209601.116 PMC5340976

[32] Patro, R., Duggal, G., Love, M. I., Irizarry, R. A. & Kingsford, C. Salmon provides fast and bias-aware quantification of transcript expression. *Nat. Methods* **14**, 417–419 (2017). 28263959 10.1038/nmeth.4197 PMC5600148

[33] Powell, D. Drpowell/Degust 4.1.1. *Zenodo* https://zenodo.org/records/3501067 (2019).

[34] Law, C. W., Chen, Y., Shi, W. & Smyth, G. K. Voom: precision weights unlock linear model analysis tools for RNA-seq read counts. *Genome Biol.* **15**, R29 (2014). 24485249 10.1186/gb-2014-15-2-r29 PMC4053721

[35] Wu, T. et al. Clusterprofiler 4.0: a universal enrichment tool for interpreting omics data. *Innovation* **2**, 100141 (2021). 34557778 10.1016/j.xinn.2021.100141 PMC8454663

[36] Coleman, M. D. in *Anesthesia Secrets* 4th edn (ed. Duke, J.) 17–23 (Mosby, 2011).

[37] Vento, M. et al. Preterm resuscitation with low oxygen causes less oxidative stress, inflammation, and chronic lung disease. *Pediatrics* **124**, e439–e449 (2009). 19661049 10.1542/peds.2009-0434

[38] Kapadia, V. S. et al. Resuscitation of preterm neonates with limited versus high oxygen strategy. *Pediatrics* **132**, e1488–e1496 (2013). 24218465 10.1542/peds.2013-0978 PMC3838529

[39] Badurdeen, S. et al. Rapid oxygen titration following cardiopulmonary resuscitation mitigates cerebral overperfusion and striatal mitochondrial dysfunction in asphyxiated newborn lambs. *bioRxiv*10.1101/2024.07.27.603805 (2024). 10.1177/0271678X241302738 PMC11584996 39576879

[40] Kapadia, V. & Oei, J. L. Optimizing oxygen therapy for preterm infants at birth: are we there yet?. *Semin. Fetal Neonatal Med.* **25**, 101081 (2020). 32044281 10.1016/j.siny.2020.101081 PMC8958722

[41] Rantakari, K. et al. Early oxygen levels contribute to brain injury in extremely preterm infants. *Pediatr. Res.* **90**, 131–139 (2021). 33753894 10.1038/s41390-021-01460-3 PMC7984503

[42] Dekker, J. et al. The effect of initial high vs. low Fio(2) on breathing effort in preterm infants at birth: a randomized controlled trial. *Front. Pediatr.* **7**, 504 (2019). 31921719 10.3389/fped.2019.00504 PMC6927294

[43] Blank, D. A. et al. Lung aeration reduces blood pressure surges caused by umbilical cord milking in preterm lambs. *Front. Pediatr.* **11**, 1073904 (2023). 37025294 10.3389/fped.2023.1073904 PMC10071016

[44] Lakshminrusimha, S., Vali, P., Chandrasekharan, P., Rich, W. & Katheria, A. Differential alveolar and systemic oxygenation during preterm resuscitation with 100% oxygen during delayed cord clamping. *Am. J. Perinatol.* **40**, 630–637 (2021). 34062568 10.1055/s-0041-1730362 PMC11870811

[45] Hillman, N. H. et al. Inhibitors of inflammation and endogenous surfactant pool size as modulators of lung injury with initiation of ventilation in preterm sheep. *Respir. Res.* **11**, 151 (2010). 21034485 10.1186/1465-9921-11-151 PMC2978154

[46] Hillman, N. H. et al. Brief, large tidal volume ventilation initiates lung Injury and a systemic response in fetal sheep. *Am. J. Respir. Crit. Care Med.* **176**, 575–581 (2007). 17641159 10.1164/rccm.200701-051OC PMC1994225

[47] Kotecha, S., Wilson, L., Wangoo, A., Silverman, M. & Shaw, R. J. Increase in interleukin (Il)-1β and Il-6 in bronchoalveolar lavage fluid obtained from infants with chronic lung disease of prematurity. *Pediatr. Res.* **40**, 250–256 (1996). 8827773 10.1203/00006450-199608000-00010

[48] Willet, K. E. et al. Intra-amniotic injection of Il-1 induces inflammation and maturation in fetal sheep lung. *Am. J. Physiol. Lung Cell. Mol. Physiol.* **282**, L411–L420 (2002). 11839534 10.1152/ajplung.00097.2001

[49] Ikegami, T. et al. Effects of intrauterine Il-6 and Il-8 on the expression of surfactant apoprotein mRNAs in the fetal rat lung. *Eur. J. Obstet. Gynecol. Reprod. Biol.* **93**, 97–103 (2000). 11000512 10.1016/s0301-2115(00)00247-5

[50] Hirani, D. et al. Macrophage-derived Il-6 trans-signalling as a novel target in the pathogenesis of bronchopulmonary dysplasia. *Eur. Respir. J.* **59**, 2002248 (2022). 10.1183/13993003.02248-2020 PMC8850688 34446466

[51] Hillman, N. H. et al. Inflammation and lung maturation from stretch injury in preterm fetal sheep. *Am. J. Physiol. Lung Cell Mol. Physiol.* **300**, L232–L241 (2011). 21131401 10.1152/ajplung.00294.2010 PMC3043810

[52] Wallace, M. J. et al. Early biomarkers and potential mediators of ventilation-induced lung injury in very preterm lambs. *Respir. Res.* **10**, 19 (2009). 19284536 10.1186/1465-9921-10-19 PMC2662809

[53] Wright, C. J. & Dennery, P. A. Manipulation of gene expression by oxygen: a primer from bedside to bench. *Pediatr. Res.* **66**, 3–10 (2009). 19287338 10.1203/PDR.0b013e3181a2c184 PMC2700778

